# Diagnosis and classification of pediatric acute appendicitis by artificial intelligence methods: An investigator-independent approach

**DOI:** 10.1371/journal.pone.0222030

**Published:** 2019-09-25

**Authors:** Josephine Reismann, Alessandro Romualdi, Natalie Kiss, Maximiliane I. Minderjahn, Jim Kallarackal, Martina Schad, Marc Reismann

**Affiliations:** 1 Department of Pediatric Surgery, Charité –Universitätsmedizin Berlin, Augustenburger Platz, Berlin, Germany; 2 OakLabs GmbH, Hennigsdorf, Germany; Medical University of Vienna, AUSTRIA

## Abstract

Acute appendicitis is one of the major causes for emergency surgery in childhood and adolescence. Appendectomy is still the therapy of choice, but conservative strategies are increasingly being studied for uncomplicated inflammation. Diagnosis of acute appendicitis remains challenging, especially due to the frequently unspecific clinical picture. Inflammatory blood markers and imaging methods like ultrasound are limited as they have to be interpreted by experts and still do not offer sufficient diagnostic certainty. This study presents a method for automatic diagnosis of appendicitis as well as the differentiation between complicated and uncomplicated inflammation using values/parameters which are routinely and unbiasedly obtained for each patient with suspected appendicitis. We analyzed full blood counts, c-reactive protein (CRP) and appendiceal diameters in ultrasound investigations corresponding to children and adolescents aged 0–17 years from a hospital based population in Berlin, Germany. A total of 590 patients (473 patients with appendicitis in histopathology and 117 with negative histopathological findings) were analyzed retrospectively with modern algorithms from machine learning (ML) and artificial intelligence (AI). The discovery of informative parameters (biomarker signatures) and training of the classification model were done with a maximum of 35% of the patients. The remaining minimum 65% of patients were used for validation. At clinical relevant cut-off points the accuracy of the biomarker signature for diagnosis of appendicitis was 90% (93% sensitivity, 67% specificity), while the accuracy to correctly identify complicated inflammation was 51% (95% sensitivity, 33% specificity) on validation data. Such a test would be capable to prevent two out of three patients without appendicitis from useless surgery as well as one out of three patients with uncomplicated appendicitis. The presented method has the potential to change today’s therapeutic approach for appendicitis and demonstrates the capability of algorithms from AI and ML to significantly improve diagnostics even based on routine diagnostic parameters.

## Introduction

Acute appendicitis is one of the most common causes for emergency surgery with a lifetime risk between 7 and 9% in industrialized countries. Especially children are affected with a peak incidence in adolescence [1]. Correct diagnosis of appendicitis is still a challenge. Especially clinical decision making is difficult due to great differences between investigators. Published sensitivity values for frequently favored clinical signs like right lower quadrant pain vary between 49% (specificity 73%) and 69% (specificity 61%) [2, 3].

Although individual concepts vary, there is broad consensus on basic diagnostic measures in cases of suspected acute appendicitis. Suspicion of acute appendicitis is usually based on clinical presentation and patient's history. Further laboratory diagnostics include white blood cell counts, absolute neutrophil count and C-reactive protein (CRP). Routine diagnostic is usually completed by imaging studies like ultrasound, computed tomography or magnetic resonance imaging [4].

Single laboratory values such as neutrophil and leucocyte counts as well as increased C-reactive protein (CRP) provide diagnostic value: sensitivities for the latter range between 38 and 70% (specificities 85 and 65%, respectively) [5, 6]. In two recent publications including the herein reported 590 patients we analyzed possible constitutive differences between complicated and uncomplicated appendicitis with regard to cellular subpopulations in white blood cell counts and CRP: Significant and time stable differences were found [7, 8]. Especially relative eosinophilia in patients with uncomplicated appendicitis was remarkable. The investigation did not go beyond a statistical analysis of the individual parameters and the discriminatory capacity of the single parameters was low.

The appendiceal diameter, an unbiased and even in children age-independent measurement value, has previously shown to provide a high sensitivity to diagnose appendicitis in adults with an accuracy of 79% [6, 7]. In a recently published study, including the 590 patients of the present study, we have shown that ultrasound has also value for the differentiation of complicated from uncomplicated appendicitis [9]. The appendix could be sonographically visualized in a clear majority of 862 out of 1017 included patients (85%). Other parameters such as blood values were not the subject of the statistical analysis.

Further ambitions of improving the diagnosis of appendicitis in adults focus on modeling multiple parameters, including clinical and laboratory ones [10].

However, in children, especially with an age below six years, clinical signs and symptoms are less reliable. Clinically complicated appendicitis in this age group is frequently hard to differentiate—especially from gastroenteritis [11].

Clinically based scores such as the Alvaro Score and the Pediatric Appendicitis Score (PAS) have been described as potential tools for identifying children with appendicitis. However, reported sensitivities and respective specificities are marked by an extensive variability [11]. A key problem is probably the low interobserver reliability of predictor variables [10].

The aim of the present study was to establish a model for decision making for suspected acute appendicitis in children, which is based on reliable non-clinical parameters unbiased from interpretation or expert opinion: counts of cell types in whole blood, CRP values and the appendiceal diameter as a simple sonographic numerical measure.

A special focus was the differentiation between uncomplicated (phlegmonous) and complicated (gangrenous/perforated) appendicitis. Early diagnosis of complicated inflammation is particularly important, because this severe type of disease primarily requires surgical treatment. In contrast, for uncomplicated appendicitis conservative strategies are under investigation and will most probably be primarily applied in the near future, as shown by a current multicenter randomized controlled trial [12].

Though correlations between cellular compartments in full blood and the type of disease have been shown previously [7, 8], the diagnosis of complicated appendicitis remains challenging. With the study we also strived to demonstrate the feasibility of a multi-parameter model for the differential diagnosis of appendicitis.

## Materials and methods

### Study population

We present a single-center, retrospective study of patients aged 0–17 years who underwent surgery for suspected acute appendicitis at the Department of Pediatric Surgery of Charité - Universitätsmedizin Berlin between December 2006 and September 2016. The study was approved by our institutional review board and the ethical committee (reference number EA2/169/18).

Medical charts of all patients who were operated for suspected acute appendicitis were reviewed for gender, age and standard diagnostic parameters: CRP values, cell counts in full blood, histopathologies and ultrasound findings. Exclusion criteria were missing histopathologies or laboratory values, concomitant chronic disease, secondary or elective appendectomy and other pathologies of the appendix like oxyuriasis and carcinoid.

### Histopathological classification

Histopathological analyses were retrospectively reviewed to classify the patients into three groups: uncomplicated (phlegmonous) appendicitis, complicated (gangrenous/perforated) appendicitis and normal appendix (negative for appendicitis). In clinical settings the histological finding of phlegmonous appendicitis is associated with uncomplicated courses (UA), whereas gangrenous appendicitis and perforation are categorized as acute complicated appendicitis (CA) [13, 14]. Uncomplicated phlegmonous appendicitis was defined by transmural neutrophilic infiltration of the appendix without signs of gangrene or perforation. Gangrenous appendicitis was characterized by ischemic areas leading to transmural myonecrosis leading possibly to perforation with presence of a transmural defect [15].

### Laboratory data

Routinely performed white blood cell counts included the following mature leukocyte subpopulations: eosinophil granulocytes (eosinophils), neutrophil granulocytes (neutrophils), lymphocytes, basophilic granulocytes (basophils), and monocytes supplemented by thrombocytes and C-reactive protein (CRP) at time of hospital admission.

### Sonography

**Fig 1:**
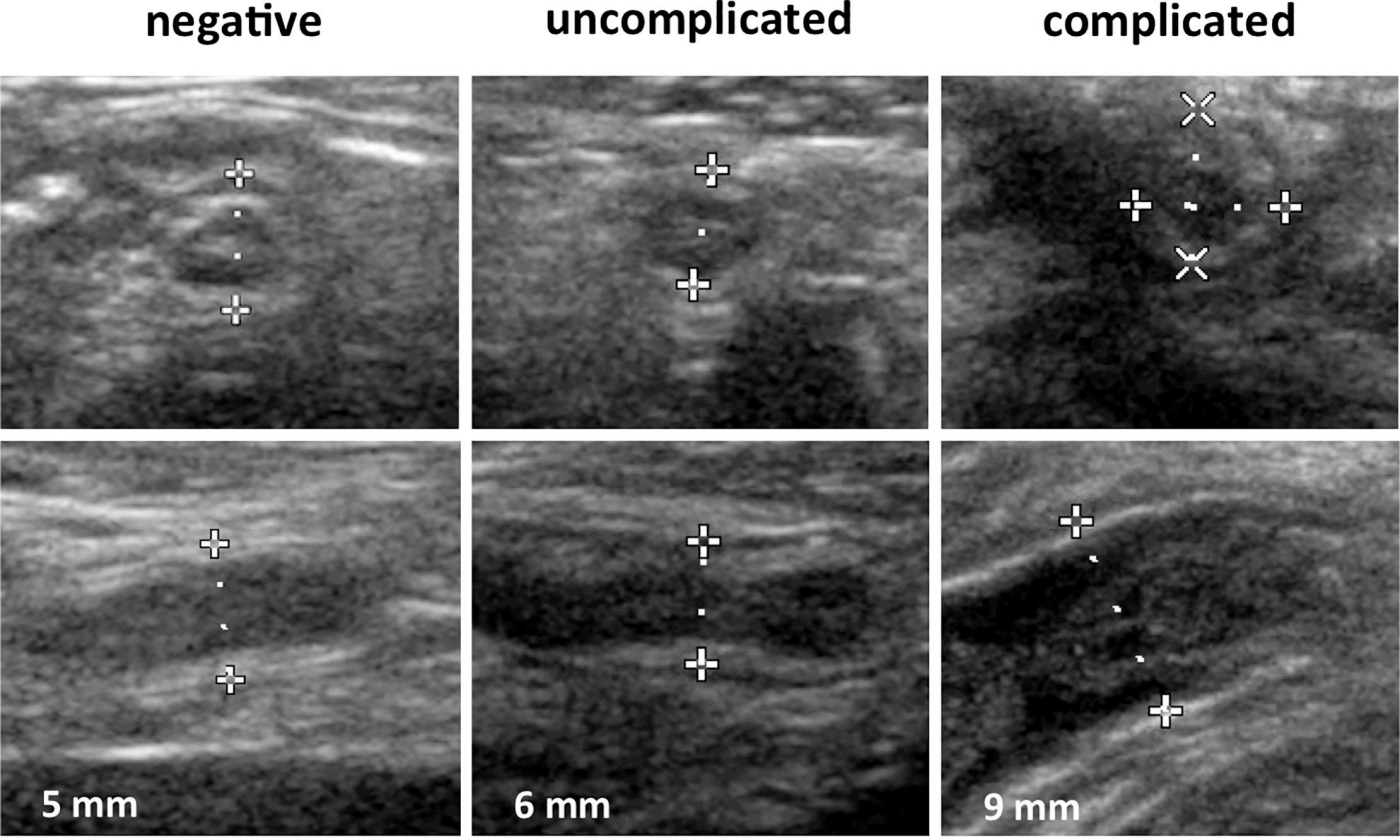
Sonographic images of appendices from 8 years old female patients without inflammation, with uncomplicated and with complicated appendicitis; cross and longitudinal sections, respective maximum diameters [mm].

All included sonographic measures had been routinely performed by pediatric radiologists within the first presentation of the patients in the emergency department. All reported ultrasound examinations were performed or directly supervised by four experienced consultant pediatric radiologists with sonographic experience of at least 19 up to 37 years. The appendix was measured from outer wall to outer wall [16, 17]. Fig 1 shows exemplary pictures of appendices without and with uncomplicated phlegmonous as well as with complicated gangrenous appendicitis.

### Development and validation of biomarker signatures for diagnosis of appendicitis

A supervised learning algorithm is used to analyze laboratory data and to build a prediction model for diagnosis of appendicitis based on relevant biomarkers. This is a two-step process summarized as *discovery* and *validation*. The model building and biomarker selection was performed on a portion of the available sample data denominated as “discovery set”; the performance of the final model was measured in a distinct data set denominated as “validation”. The input data consisted of *n* samples, each described by a set of *p* variables, represented by the biomarker values. Concretely, we had a data matrix X consisting of *n* lines and *p* columns.

In the discovery phase, we identified relevant biomarkers: we first built a sequence of distinct biomarker signatures {bm_1_, ….,bm_j_,…, bm_m_} and then implement a binary classification problem fitting the parameters of a linear model on the discovery data X_discovery_, whose columns *p*_*bmj*_ were filtered according to the biomarker signatures. The parameters of the linear model were optimized with the Limited-memory BFGS (LBFGS) algorithm [18]. Since the two classes ('complicated' + 'uncomplicated'/ 'negative') were highly imbalanced with respect to sample sizes, the learning mistakes relative to the class with larger sample number were penalized with a weight coefficient during the optimization process. In this way, the quality of each biomarker signature was measured with the cross-validation accuracy on the discovery data.

All performance values denoted here were obtained measuring the performance of the trained obtained model, on the validation data X_validation_.

Out of a total of 1102 patients, 590 patients with availability of the required histological, laboratory and sonographic parameters were used for the discovery and validation process. We aimed to selectively investigate the influence of the sonographic parameter within the signature. For diagnosing appendicitis, 390 patients were used for validation of the signature, which was exclusively based on lab parameters, while 350 patients were used for validation of the signature with the additional parameter from sonography. For the differentiation between complicated and uncomplicated appendicitis, 298 patients were used for validation. The validation set contained a portion of patients which were diagnosed negative for appendicitis to take the false positive rate of the signature for diagnosing appendicitis into account and simulate real-world clinical practice: The complicated appendicitis must be discriminated from uncomplicated appendicitis and negative findings.

**Fig 2:**
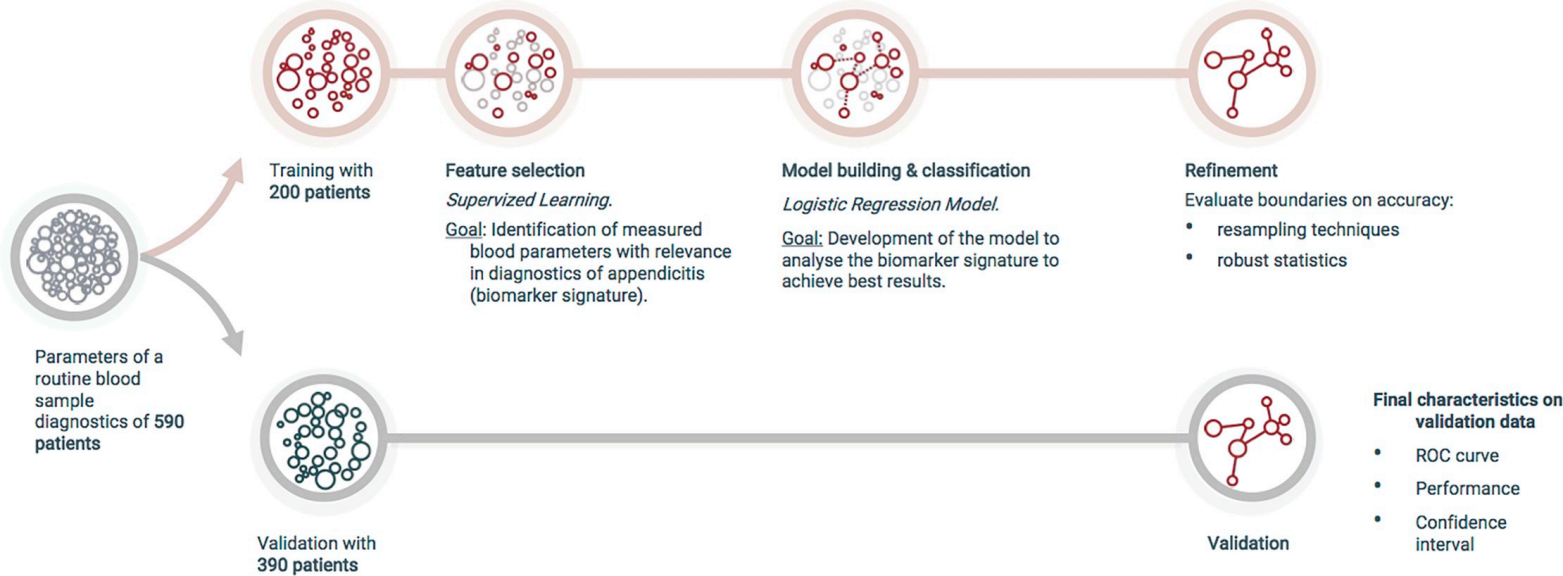
Illustration of development and validation of biomarker signatures.

Fig 2 illustrates the development and validation of biomarker signatures for the example of appendicitis diagnostics. Respective patient numbers and epidemiological data are shown in Tables 1 and 2.

**Table 1:** Numbers and characteristics of patients for development of specific biomarker signatures: Diagnosis of acute appendicitis.

	number	age [years]	gender ♂ / ♀ [total no / %]	negative [total no / %]	uncomplicated [total no / %]	complicated [total no / %]
discovery	200	10.2 ± 4.4	103 (51.5%) / 97 (48.5%)	59 (29.5%)	76 (38%)	65 (32.5%)
validation	390	10.7 ± 3.1	221(56.6%) / 169 (43.3%)	58 (14.9%)	214 (54.9%)	118 (30.2%)
total	590	10.5 ± 3.6	323 (54.7%) / 267 (54.3%)	117 (19.8%)	290 (49.2%)	183 (31%)

**Table 2:** Numbers and characteristics of patients for development of specific biomarker signatures: Detection of complicated appendicitis.

	number	age [years]	gender ♂ / ♀ [total no / %]	negative [total no / %]	uncomplicated [total no / %]	complicated [total no / %]
discovery	192	9.1 ± 3.6	109 (56.8%) / 83 (43.2%)	-	101 (52.6%)	91 (47.4%)
validation	298	10.9 ± 3.2	173 (58%) / 125 (42%)	21 (7%)	186 (62.4%)	91 (30.5%)
total	490	10 ± 4.8	283 (57.8%) / 207 (42.2%)	21 (4.3%)	287 (58.5%)	182 (37.1%)

### ROC analysis

Once the best model has been defined with the fitted coefficients, it can be used to predict the *diagnostic status* of a patient with class probability [19].

The output class probabilities may be interpreted as different separation thresholds between class prediction. Each threshold is a trade-off for the model to predict a number of true/false positives and true/false negatives.

The diagnostic ability of the model (sensitivity and specificity) was tested on the validation set, counting the predicted true positive/false positive rate at different thresholds. The result is illustrated with a Receiver Operating Characteristic (ROC) plot.

Within the ROC analysis, the results of the signatures were compared to those of established laboratory parameters: CRP, leukocyte and neutrophil counts. For comparision, cut-off points were selected which we considered to be of clinical interest [20], that is a sensitivity above 90%. This was reached at the cut-off point of 67% specificity for the diagnosis of acute appendicitis and of 33% specificity for differentiation between complicated and uncomplicated appendicitis. Errors were calculated performing bootstrap resampling.

## Results

The distribution of analyzed values for whole blood cell counts, CRP and appendiceal diameter differed between patients with and without appendicitis and between patients with complicated and uncomplicated inflammation, respectively (S1 Fig).

Based on the ten parameters CRP, thrombocytes, leukocytes, neutrophils, eosinophils, basophils and immature granulocytes, lymphocytes and monocytes as well as the appendiceal diameter, two biomarker signatures were developed containing the most informative parameters to diagnose appendicitis and complicated appendicitis, respectively.

For the diagnosis of appendicitis, a selective biomarker signature was developed containing basophils, leukocytes, monocytes, neutrophils, CRP and the appendiceal diameter. For the differential diagnosis of complicated versus uncomplicated appendicitis, a selective biomarker signature was developed including basophils, eosinophils, monocytes, thrombocytes, CRP, supplemented by the appendiceal diameter.

**Fig 3:**
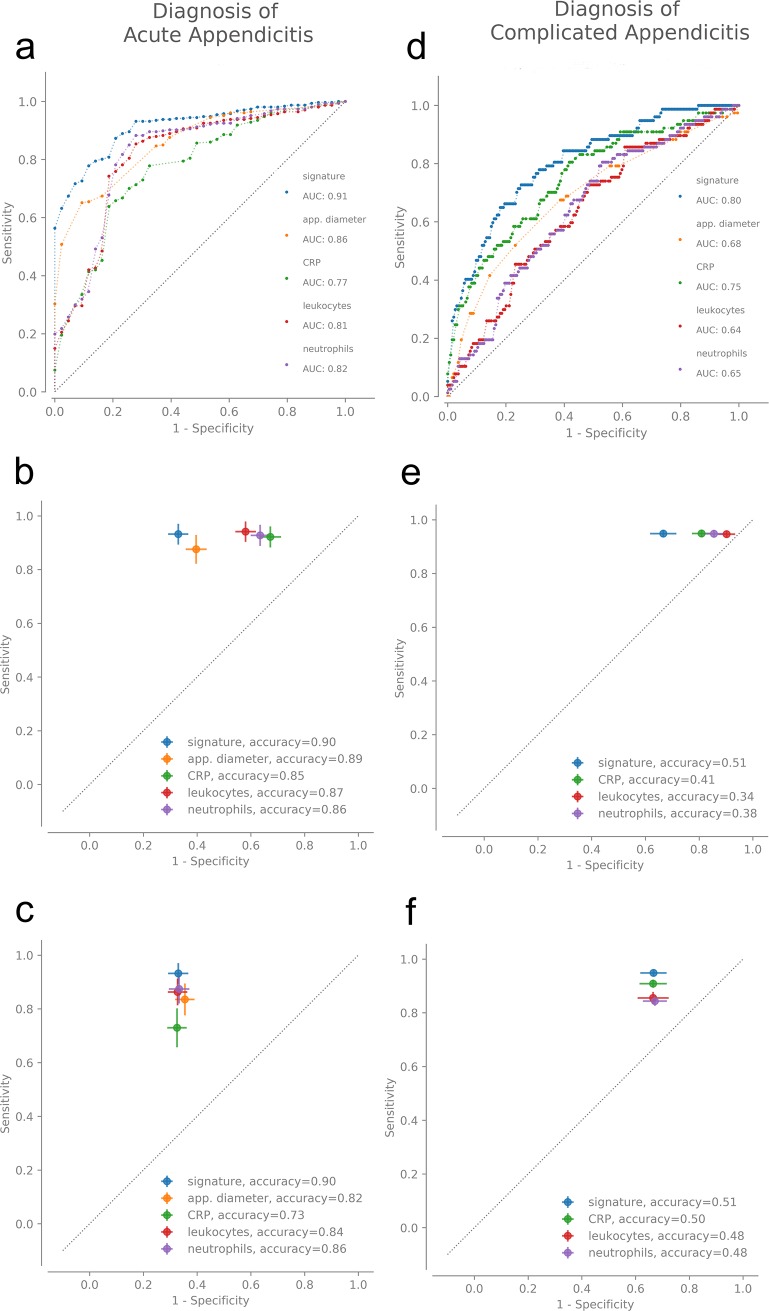
ROC curves. a: analysis of the predictive capacity for discrimination between appendicitis and normal appendix (biomarker signature vs. conventional values CRP, neutrophils, leukocytes and appendiceal diameter). b and c: best cut-off biomarker signature vs. respective sensitivities (b) and specificities (c) of conventional lab values. d: analysis of the diagnostic capacity for discrimination between complicated and uncomplicated appendicitis (biomarker signature vs. conventional values CRP, neutrophils and leukocytes). e and f: best cut-off biomarker signature vs. respective sensitivities (e) and specificities (f) of conventional values. AUCs and accuracies are shown in Tables 3 and 4.

The diagnostic capacities of the developed biomarker signatures were compared to single widely accepted values for diagnostics of acute appendicitis: CRP, leukocytes, neutrophils and appendiceal diameter. Fig 3 shows the results of the respective analysis. ROC curves for diagnosis of acute appendicitis and complicated appendicitis demonstrate increased areas under the curve (AUCs) (Fig 3A and 3D). At selected cut-off points, the properties of the biomarker signatures were compared to those for CRP, leukocytes and neutrophils (Fig 3B, 3C, 3E and 3F). For both diagnostic applications the properties of the biomarker signatures outperform those of the conventional single lab values. Tables 3 and 4 show the exact values for AUCs as well as properties at the cut-off points.

**Table 3:** Areas under the curve (AUC) of ROC curve shown in Fig 3A; accuracies of biomarker signatures and of conventional single markers with respect to sensitivity and specificity levels at selected points for diagnosis of an acute appendicitis with the biomarker signature (sensitivity 0.93, specificity 0.67; Fig 3A–3C).

Biomarker	AUC (Fig 3A)	Specificity at targeted sensitivity of 0.93 [95% CI] (Fig 3B)	Accuracy at targeted sensitivity of 0.93 (Fig 3B)	Sensitivity at targeted specificity of 0.67 [95% CI] (Fig 3C)	Accuracy at targeted specificity of 0.67 (Fig 3C)
signature	0.91	0.67 [0.59–0.74]	0.90	0.93 [0.85–1.00]	0.90
appendiceal diameter	0.86	0.61 [0.53–0.67]	0.89	0.83 [0.72–0.94]	0.82
CRP	0.77	0.33 [0.24–0.40]	0.85	0.73 [0.59–0.85]	0.73
leukocytes	0.81	0.42 [0.35–0.5]	0.87	0.86 [0.76–0.94]	0.84
neutrophils	0.82	0.37 [0.30–0.41 ]	0.86	0.87 [0.76–0.97]	0.86

**Table 4:** Areas under the curve (AUC) of ROC curve shown in Fig 3D; accuracies of biomarker signatures and of conventional single markers with respect to sensitivity and specificity levels at selected points for differentiation from complicated appendicitis with the biomarker signature (sensitivity 0.95, specificity 0.33; Figs d-f).

Biomarker	AUC (Fig 3D)	Specificity at targeted sensitivity of 0.95 [95% CI] (Fig 3E)	Accuracy at targeted sensitivity of 0.95 (Fig 3E)	Sensitivity at targeted specificity of 0.33 [95% CI] (Fig 3F)	Accuracy at targeted specificity of 0.33 (Fig 3F)
signature	0.80	0.33 [0.24,-0.42]	0.51	0.95 [0.93–0.97]	0.51
CRP	0.75	0.19 [0.12–0.26]	0.41	0.91 [0.88–0.93]	0.5
leukocytes	0.64	0.1 [0.3–0.16]	0.34	0.86 [0.82–0.90]	0.48
neutrophils	0.65	0.14 [0.8–0.24]	0.38	0.84 [0.81–0.88]	0.48

While the appendiceal diameter is fundamental for the diagnostic ability of the analyzed signature (AUC 0.9 with appendiceal diameter vs. 0.8 without), the diameter did not significantly alter the diagnostic capacity for differentiation of complicated appendicitis (AUC 0.81 vs. 0.80) (S2 Fig). Apparently, this sonographic parameter does not reveal a significant predictive capacity as soon as appendicitis has been diagnosed.

## Discussion

Though appendicitis is one of the major causes for emergency surgery, its correct diagnosis remains challenging. In this study, we have developed a biomarker signature based on routine unbiased parameters that is capable of becoming the gold standard for the diagnosis of appendicitis. A second objective was to demonstrate that a multi-parameter model is capable of discriminating between complicated and uncomplicated appendicitis. This is a prerequisite for establishing a modern medical treatment for appendicitis to the patients´ benefit.

To avoid methodological weaknesses, we rigorously separated the discovery set from the validation set to be able to determine the value of the outcome. We have chosen a linear model as the initial histograms revealed that the data is linearly separable. A more complex model would potentially fit an excess of parameters to the specifics of the one clinical site which, in turn, would result in low reproducibility. Out of all measured values, two biomarker signatures with clinical relevance were selected by the linear model for discriminating patients with appendicitis from those without, and of patients with complicated from those with uncomplicated inflammation. We have compared the results of the biomarker signatures with the widely used inflammatory values white blood cell counts, CRP and leucocytes within our model: Sensitivities, specificities, accuracies and AUCs of the traditional values were exceeded by those of the linear model.

Imaging techniques are most valuable when acute appendicitis is suspected and have been described as superior to patient history, physical examination, laboratory findings or scores [10, 21]. Regarding sonography, the appendiceal diameter is a very useful discriminating parameter [16, 17]. Furthermore, it is largely independent of personal interpretation. We confirmed the independence of the appendiceal diameter from the age as described previously [16] and included this parameter as an input variable into the linear model. Out of the cellular subpopulations in the white blood cell counts and the appendiceal diameters of the included patients a biomarker signature was developed. At a specificity of 67% and a sensitivity of 93%, an accuracy of 90% is reached on validation data. Such a diagnostic test could prevent two of three patients without appendicitis from appendectomy.

The superiority of our approach for diagnosing appendicitis is reached by combining complementary methods: lab measured values and a value measured by a radiologist, the appendiceal diameter. In contrast, for ruling out complicated appendicitis, the appendicitis diameter is not vital in our model. Using lab measured values only, the model reached a sensitivity of 95% at a specificity of 33% demonstrating the capability of the model to rule out complicated appendicitis in one out of three cases and thus avoiding surgery.

A few studies describe decision making within diagnostics for appendicitis with artificial neural networks (ANN) and achieve impressive results, e.g. 91% sensitivity with a specificity of 85% [22] and 100% sensitivity with a specificity of 97% [23]. However, both studies have serious weaknesses. A central concern is overfitting: Neural networks tend to overfit the data [20]. Even small neural networks are comprised of several weighting parameters. The above mentioned studies either report their performance without mentioning the size of the training and validation data or the reported training data is far to small to reliably fit all parameters of the neural network. Furthermore, they are characterized by inadequate description of predictor variables and absence of reproducibility testing of predictor variables as the variables “vomiting” and right lower quadrant (RLQ) tenderness and rebound pain exemplary demonstrate. “Vomiting” has been inadequately qualified binarily with “yes”or “no”in the studies and specification is missing in respect to quality, volume or number of episodes is missing. For RLQ, the determination of the interobserver reliability is missing which is extremely important, especially for children [10, 11].

A limitation of the present study is given by its retrospective design. We compensate this deficit by the exclusive inclusion of numeric data, which are essentially not due to personal interpretation. The appendiceal diameter is no exception here as it is the simplest sonographic parameter in suspected appendicitis with a high concordance rate between radiologists [9].

## Conclusions

An interdisciplinary team of physicians, life scientists and physicists presents a model for diagnosing acute appendicitis in childhood and adolescence which has the potential to establish as a gold standard. Central quality features are given by effective methodological measures especially in order to avoid overfitting and by the use of numerical parameters, which are as far as possible not prone to personal interpretation. Due to the retrospective nature of our study we do not present a ready-to-use clinical algorithm, but our approach demonstrates significant improvements compared to today's diagnosis and enables secure translation into clinical practice. Our approach also demonstrates significant value in ruling out complicated appendicitis with high sensitivity. Investigations on the OMICs level such as genome-wide gene expression profiling of specific cell compartments could be a path to increase the specificity.

## Supporting information

S1 Fig Relative distributions of values of features of the signature for a) the diagnosis of appendicitis and b) the differentiation in complicated and uncomplicated appendicitis.
(TIF)

S2 Fig Biomarker signatures for diagnosis of acute appendicitis (a) and complicated appendicitis (b) with and without inclusion of appendiceal diameter.
(TIF)
