# Supplementary figures and images for: Diagnosis and classification of pediatric acute appendicitis by artificial intelligence methods: An investigator-independent approach
Source: PLoS One. 2019 Sep 25;14(9):e0222030. doi: 10.1371/journal.pone.0222030 (PMC6760759; doi:10.1371/journal.pone.0222030)

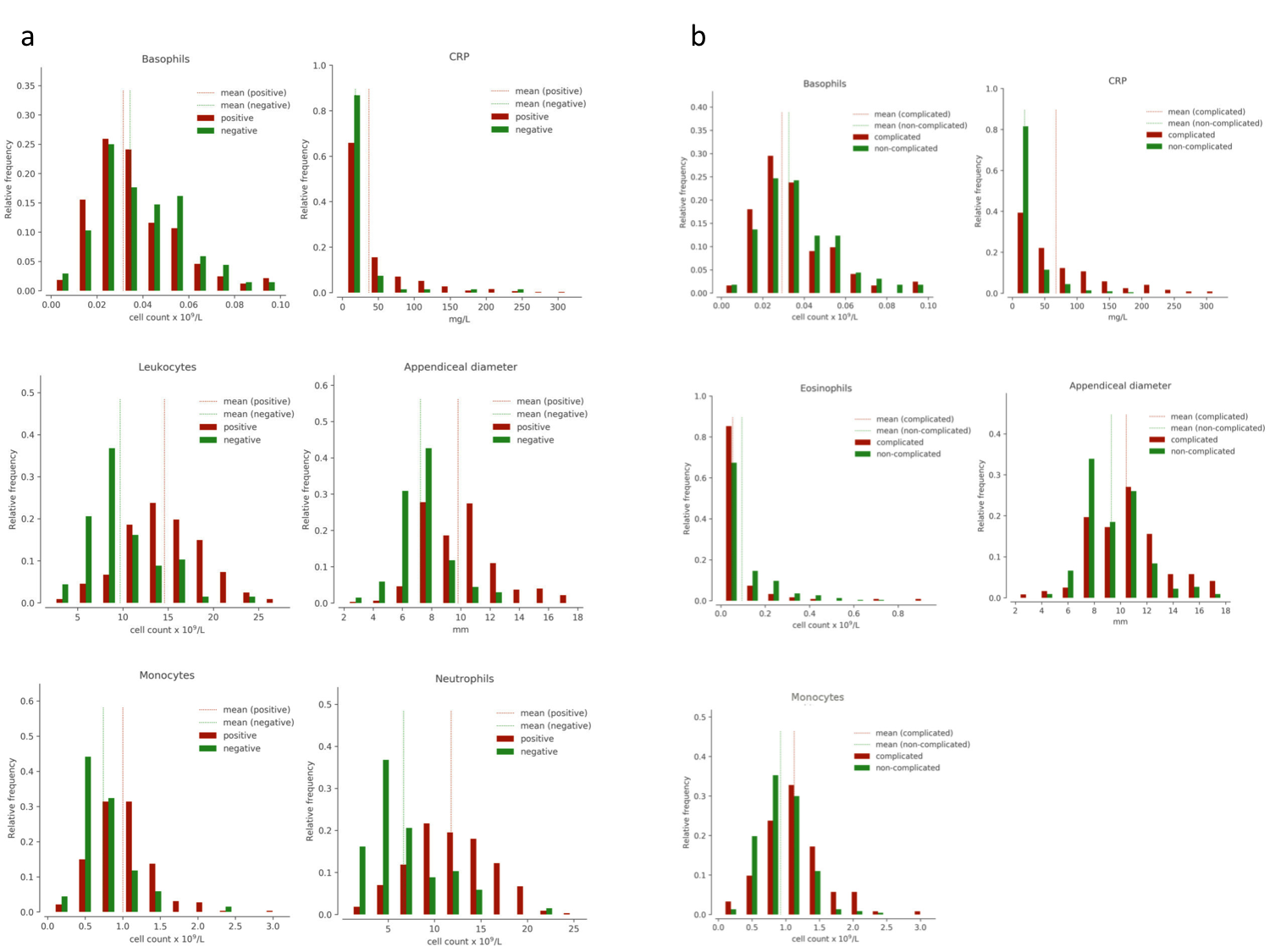

Supplement: S1 Fig — Relative distributions of values of features of the signature for a) the diagnosis of appendicitis and b) the differentiation in complicated and uncomplicated appendicitis. (TIF) [file pone.0222030.s001.tif]

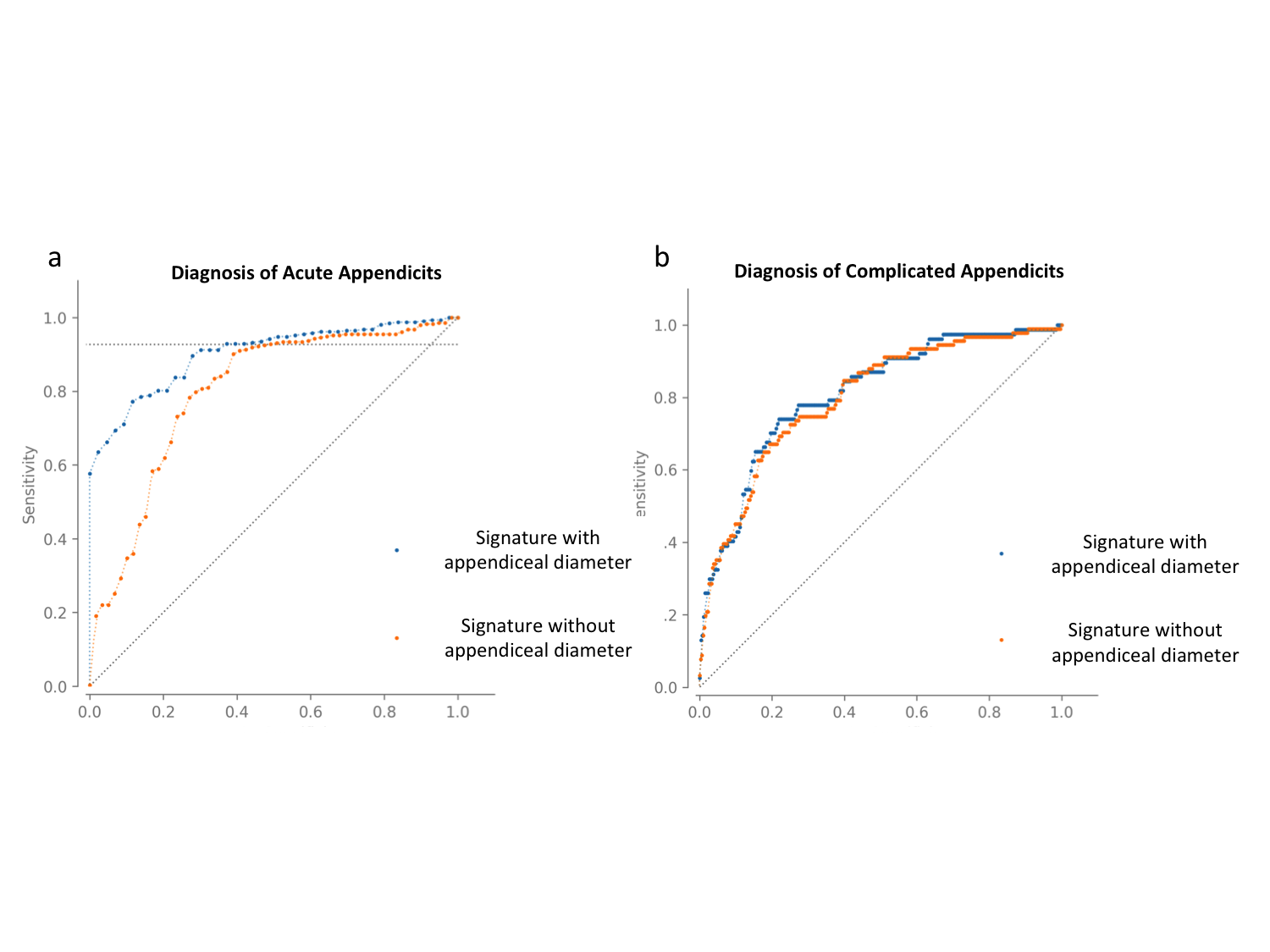

Supplement: S2 Fig — Biomarker signatures for diagnosis of acute appendicitis (a) and complicated appendicitis (b) with and without inclusion of appendiceal diameter. (TIF) [file pone.0222030.s002.tif]
